# Marek’s Disease Virus Modulates T Cell Proliferation *via* Activation of Cyclooxygenase 2-Dependent Prostaglandin E2

**DOI:** 10.3389/fimmu.2021.801781

**Published:** 2021-12-22

**Authors:** Nitin Kamble, Angila Gurung, Benedikt B. Kaufer, Ansar Ahmed Pathan, Shahriar Behboudi

**Affiliations:** ^1^ The Pirbright Institute, Woking, United Kingdom; ^2^ Department of Life Sciences, College of Health and Life Sciences, Brunel University, London, United Kingdom; ^3^ Institut für Virologie, Freie Universität Berlin, Berlin, Germany; ^4^ Faculty of Health and Medical Sciences, School of Veterinary Medicine, University of Surrey, Guildford, United Kingdom

**Keywords:** Marek’s disease virus, T cell proliferation, COX2 = cyclooxygenase 2, PGE2, immunosuppression

## Abstract

Marek’s disease virus (MDV), an avian alphaherpesvirus, infects chickens, transforms CD4+ T cells, and induces immunosuppression early during infection. However, the exact mechanisms involved in MDV-induced immunosuppression are yet to be identified. Here, our results demonstrate that MDV infection *in vitro* and *in vivo* induces activation of cyclooxygenase-2 (COX-2) and production of prostaglandin E2 (PGE2). This exerts its inhibitory effects on T cell proliferation at day 21 post infection *via* PGE2 receptor 2 (EP2) and receptor 4 (EP4). Impairment of the MDV-induced T cell proliferation was associated with downregulation of IL-2 and transferrin uptake in a COX-2/PGE2 dependent manner *in vitro*. Interestingly, oral administration of a COX-2 inhibitor, meloxicam, during MDV infection inhibited COX-2 activation and rescued T cell proliferation at day 21 post infection. Taken together, our results reveal a novel mechanism that contributes to immunosuppression in the MDV-infected chickens.

## Introduction

Several viruses that establish chronic infections have evolved mechanisms to escape immune control. In parallel, the host has developed strategies to suppress antiviral immunity to limit T cell-mediated immunopathology. Development of T cell immunity is an indispensable part of host defense against invading viruses. Viruses counteract the development of adaptive immunity through various mechanisms. One of them is the suppression of antigen specific as well as non-specific T cell proliferation into effector cells which is an essential part of adaptive immune response against infectious agent (1, 2).

Prostaglandin E2 (PGE2) is one of the prostanoid and it is involved in many physiological processes by binding to PGE2 receptors (EP1-EP4). In cells, arachidonic acid can be transformed into PGE2 by actions of cyclooxygenase (COX) enzymes and terminal prostaglandin E synthases (PGES) (3). In mammals, PGE2 exerts diverse physiological and immunological activities, including inhibitory effects on T cell functions such as T cell differentiation, proliferation, cytotoxicity, and cytokine production (3–9). Some viral infections elevate the production of PGE2 which can regulate virus replication and modulate both anti-viral innate and adaptive immune system. The contrasting effects of PGE2 on replication of different viruses have been documented (3). However, most studies have focused on the effects of virus-induced COX-2/PGE2 pathway on virus replication (10–16). We had previously shown that *in vitro* infection of chicken embryonic fibroblasts (CEFs) with virulent MDV activates COX-2/PGE2 pathway, which is involved in MDV replication (15). However, it was unclear whether MDV can also activate COX2/PGE2 pathway *in vivo* and very little was known about the effects of MDV-induced PGE2 on function of immune cells. The role of virus-induced PGE2 on modulation of T cell response had been previously suggested (14, 17, 18) and it has been shown that the administration of COX-2 inhibitor can restore T cell proliferation in murine leukemic retrovirus model (18), suggesting that COX-2 inhibitors may be used to overcome virus-induced immunosuppression *in vivo*. Here, we hypothesized that MDV-induced PGE2 may suppress T cell proliferation during MDV infection.

MDV serotype 1 (*e.g.*, RB1B) is the causative agent of Marek’s disease (MD), an economically important chicken disease, which is characterized by lymphoma formation and immunosuppression. Rispens-CVI988 is the vaccine strain of MDV serotype 1 with an overall identical gene organization compared to the virulent strain (19–21). We had previously shown that virulent MDV, but not the vaccine strain, activates expansion of Treg cells *in vivo* (22), suggesting that there is an association between Treg cell activation and MDV pathogenesis. Considering the immunosuppressive effects of PGE2 on T cell function (17, 23–26), we hypothesized that the virulent MDV, but not vaccine strain, may exert immunosuppressive effects on T cells *via* COX-2/PGE2 pathway. Our *in vitro* results had demonstrated that virulent MDV modulates cell metabolism, including COX-2 activation, in non-transformed primary chicken embryonic fibroblasts (15, 27, 28). Here, the *in vitro* and *in vivo* results demonstrate that the virulent MDV, but not Rispens-CVI988, activates the COX-2/PGE2 pathway which modulates T cell proliferation in EP2 and EP4 dependent manner. Oral administration of a COX-2 inhibitor downregulated MDV-induced COX-2 activation and restored T cell proliferation at 21-day post infection in the MDV-infected chickens.

## Materials And Methods

### Virus Preparation

One-day-old RIR chickens were infected with 200 µl phosphate buffered saline (PBS) containing 1,000 plaque forming units (PFU) of pathogenic RB1B or vaccine strain Rispens-CVI988 (Intervet, Milton Keynes, UK) *via* the intra-abdominal route. Splenocytes were harvested at 14 dpi and were co-cultured with primary chicken embryo fibroblasts (CEFs) cells for 7 days. Once the cytopathic effects were observed, the cell-associated MDV-infected CEF cells were further passaged two times on fresh CEFs, virus stocks were prepared, stored in medium 199 with Earle’s salts (E199) containing 10% heat inactivated-fetal calf serum (HI-FCS) and 10% dimethyl sulphoxide (DMSO).

### Ethics Statement

Animal experiments were approved by the ethical review committee at The Pirbright Institute (TPI) and the experiments were performed based on the guidelines and care approved by the UK government Home Office under project license PPL 30/3169. The personnel engaged in the procedures had acquired personal license from the UK Home Office.

### Animal Experiments

The specific pathogen free (SPF) mixed sex inbred Rhode Island Red (RIR) and line P2a (homozygous for the B19 haplotype) chickens reared at The Pirbright Institute. *In vivo* experiment comprising day-old line P2a chickens were inoculated with 200 µl of PBS containing 1000 PFU of MDV (RB1b) or vaccine (Rispens-CVI988) or mock infected-CEF cells (n = 6) *via* the intra-abdominal route. *In vivo* experiment comprising seven-day old RIR chickens were inoculated with 200 µl PBS containing 1000 PFU MDV or mock infected-CEF cells *via* intra-abdominal route, further, MDV infected chickens RIR chickens were divided into two groups: one was orally administered meloxicam (Cox-2 inhibitor) on daily basis, and second group kept as MDV infected only. For *ex vivo* assays, spleen was obtained from 3-week-old P2a and RIR chickens by cervical dislocation as mentioned in schedule 1 guidelines. The harvested spleen was collected aseptically in sterile PBS supplemented with penicillin-streptomycin solution (Microbiological Services, Pirbright Institute, Surrey, UK). Splenocytes obtained from P2a chickens and used in the *ex vivo* expression of COX-2 gene and T cell proliferation studies. Splenocytes obtained from RIR chickens were used to analyse effect of meloxicam on T cell proliferation.

### Cell Culture

265L lymphoblastoid monoclonal lymphoma cell line is derived from liver from inbred line P (MHC B19/B19) chickens infected with MDV (RB1b) at the Pirbright Institute (Gift from Professor Nair, Avian Oncogenic virus group, The Pirbright Institute). The cells were cultured in RPMI 1640 medium supplemented with 10 % HI-FCS, 10% tryptose phosphate broth (TPB), penicillin, streptomycin, 50 μM 2-mercaptoethanol (Life Technologies, Warrington, UK) and 1 mM sodium pyruvate (Sigma-Aldrich, Dorset, UK) at 41°C with 5% CO_2._

Primary chicken embryonated fibroblasts (CEFs) were used for infection and propagation of MDV. Briefly, chicken embryos from 10-day-old SPF eggs were obtained, homogenized and trypsinized (0.25% w/v) to generate single cell suspension. CEFs cells were seeded (1.5 × 10^5^ cells/ml) in 24-well plates in E199 medium supplemented with 5% fetal calf serum (FCS), 10% TPB, penicillin-streptomycin solution (Life Technologies, Warrington, UK), 2.7 % NaHCO3 (Sigma-Aldrich, Dorset, UK). After 24hrs, the CEF cells were infected with 100 PFU RB1B or Rispens-CVI988. Cell culture Supernatant was collected at 72hrs post infection, filtered through 0.2-micron filter to remove any potentially infected CEFs within the cell culture supernatant. It should be noted that MDV is a highly cell associated virus and MDV infected CEFs are unable to release the virus in the cell culture supernatant. No viral plaque was observed in the co-culture of non-infected CEFs with the cell culture supernatant at 72hpi.

### Prostaglandin E2 ELISA

PGE2 in the cell culture supernatant was analyzed using a competitive ELISA kit (R&D Systems, Abingdon, UK) according to the manufacturer’s instruction. In brief, microplates (96 wells), pre-coated with goat anti-mouse mAb, were blocked with PBS containing 3% BSA, and then incubated with the cell culture supernatant or recombinant PGE2. Anti-PGE2 mouse MAb was added, followed by HRP labelled PGE2 conjugate. After washing with PBS, HRP substrate 1-step™ Turbo TMB-ELISA substrate (Thermo Fisher Scientific, Paisley, UK) was added to the plates and incubated for 30 min. The reaction was stopped with addition of 0.16 M sulphuric acid, and the reaction was read at 450 nm (reference absorbance: 650nm). The concentration of PGE_2_ in collected supernatant was extrapolated from the standard curve generated with PGE_2_ standard (2500 pg/ml).

### Isolation of the Chicken Splenocytes

Splenocytes for *in vitro* studies were obtained from naïve RIR chickens, whereas for *in vivo* studies splenocytes were isolated from spleens of MDV infected RIR or MDV-infected line P2a chickens, as described in the results section. Briefly, splenocyte were obtained by mashing the spleens through 40-μm-pore-size Falcon cell strainers (BD Biosciences, Oxford, United Kingdom) overlaying the cells on Histopaque 1.083 (Sigma-Aldrich, Dorset, UK) for density gradient centrifugation (500g for 30 min at 4°C). The buffy coat from the interface was isolated, washed (250g for 10 min at 4°C) and suspended in complete media containing Roswell Park Memorial Institute medium-1640 (RPMI-1640) supplemented with 5% heat inactivated-foetal calf serum (HI-FCS) and penicillin-streptomycin solution. After estimation of live-dead cell number and viability by trypan blue exclusion method, the splenocytes count was adjusted to 5 x 10^6^ cells/ml. Adjusted cells were directly used or incubated in complete media containing Con-A (1µg/ml) at 41°C with 5% CO_2_ for further use.

Chicken CD4+, CD3 ^+^ or CD3^neg^ cells were purified from splenocytes using anti-phycoerythrin (PE) Micro-Bead kits as per manufacturer’s protocol (Miltenyi Biotec, Woking, UK). Briefly, chicken splenocytes (1 × 10^7^) were incubated (30 min at 4°C) with PE-conjugated mouse anti-chicken CD3 mAbs or PE-conjugated mouse anti-chicken CD4 mAb (10 μl/10^7^ cells) (Cambridge Bioscience, Cambridge, UK). The cells were centrifuged (300g for 10 min at 4°C), incubated (30 min at 4°C) with anti-PE MicroBeads (20 μl/10^7^ cells) (Miltenyi Biotec, Woking, UK). The unbound MicroBeads were removed by 2x centrifugation (300g for 10 min at 4°C) and the stained cells were subjected to MACS column (Miltenyi Biotec, Woking, UK) for isolation ofcells. The purity of the isolated CD3+ T cells, CD3^neg^ cells or CD4+ T cells was evaluated by flow cytometry and found to be >97%.

### Preparation of Chicken Bone Marrow Derived Dendritic Cells (BMDCs)

The chicken BMDCs were generated from bone marrow cells *in vitro* as described previously (29). Briefly, bone marrow cells was harvested from the femur by cutting the elongated ends and flushing the cells with PBS using 27-gauge needle. Bone marrow was mashed through 40-μm-pore-size Falcon cell strainers (BD Biosciences, Oxford, United Kingdom) to obtain single cell suspension which was overlayered on Histopaque 1.119 (Sigma-Aldrich, Dorset, UK) and subjected to density gradient centrifugation (500g for 30 min at 4°C). Buffy coat was carefully isolated from interface, washed and re-suspended in complete media supplemented with 5% chicken serum (Sigma-Aldrich, Dorset, UK), 50 μM 2-mercaptoethanol and penicillin-streptomycin solution. After estimation of live-dead cell number and viability by trypan blue exclusion method, the bone marrow cells were adjusted to 1 x 10^6^ cells/ml. For differentiation of bone marrow cells into BMDCs, the cell culture media was supplemented with chicken GM-CSF (10 ng/ml) and IL-4 (20ng/ml) (Cambridge Bioscience, Cambridge, UK). The cells were fed with fresh media and the growth factors on day 1, 3, 5 and 7 of culture. Prior to experiments, the BMDCs were gently harvested using 1x TrypLE select solution (Gibco, Paisley, UK) and seeded in desired cell density.

### Flow Cytometry

Carboxy fluorescein diacetate, succinimidyl ester (CFSE)-based T cell proliferation assay was utilized to examine the inhibitory effects of the cell culture supernatant on chicken T cell proliferation. The cells were stained with Vybrant CFDA SE cell tracer dye (Life Technologies, Warrington, UK) as recommended by the manufacturer’s instruction. Briefly, splenocytes (10 x10^6^ cells/ml) were suspended in pre-warmed PBS (37°C) and mixed with equal volume of the dye (5 µM) to give final concentration 2.5 µM of the dye. Following 15 min incubation of the cells with the dye at 41°C, the cells were washed with pre-warmed PBS, suspended in cell culture media for 30 min at 41°C, and the CFSE-stained cells were cultured in the presence of Concanavalin A (Con-A; 10 μg/mL) for 72 hrs (at 41°C with 5% CO2). In some experiments, CFSE-labelled cells were initially incubated with non-toxic concentration of SC236 (COX-2 inhibitor: 5 μg/mL), TG4-155 (EP2 antagonist: 4 μM), ER-819762 (EP4 antagonist: 8 μM) (Bio-Techne Ltd., Abingdon, UK) for 2 hrs prior to stimulation with Con-A. In some experiments, purified chicken CFSE-labelled CD3+ T cells were co-cultured with CD3^neg^ cells in the presence of Con-A (10 μg/mL) with or without addition of the chemical inhibitors of PGE_2_-COX-2 pathway. In some experiments, BMDCs were treated with PGE2 or the cell culture supernatant, washed prior to co-culture with CFSE-labelled CD3+ T cell in presence of Con-A for 72hrs. CD3+CD25+ expression was analysed by flow cytometry in splenocytes stimulated with con-A in presence of PGE2 or MDV orvaccine or CEF or 265L sup. The cells were stained with 7AAD (BD Bioscience, Oxford, UK) for dead cell exclusion before acquisition in BD LSRFortessa™ flowcytometry (BD Bioscience, Oxford, UK) and analysis of the data using FlowJo v10.07.Chemical used in this study were first evaluated for its dose dependent toxicity by 7-AAD staining and the highest, nontoxic concentrations of the inhibitors and chemicals were selected.

### qRT-PCR for COX-2 and IL2 Expression

Fold changes in mRNA level of COX-2 gene in splenocytes of MDV infected and vaccinated chickens and IL-2 gene in splenocytes (from non-infected birds) stimulated with Con-A in presence of I) MDV supernatant (10%) II) Vaccine supernatant (10%) III) control supernatant IV) 265L supernatant (10%) or V) recombinant PGE2 (10 µg/ml) were calculated by qRT-PCR. Briefly RNA was isolated by TRIzol (Life Technologies, Warrington, UK) and converted into cDNA after DNase treatment with Superscript III first-strand synthesis (Life Technologies, Warrington, UK) and used as template for qRT-PCR. List of primers are shown in **Table 1**. SYBR green chemistry based qRT-PCR was performed on Applied Biosystems 7500 qPCR system by using Luna^®^ universal qPCR master mix as per manufacturer’s protocol.

**Table 1:** List of primers used for qRT-PCR.

Target Gene	Acc. No.		Sequence (5’–3’)	Tm (°C)
**IL-2**	NM_204153.1	For	ATCTTTGGCTGTATTTCGGTAG	60
		Rev	TGGGTCTCAGTTGGTGTGTAG	
**COX-2**	NM_001167718.1	For	AGGACGGGCTATTATGGGGA	60
		Rev	GTGATCTCGACGTCAACACG	
**GAPDH**	NM_204305.1	For	GGCAGATGCAGGTGCTGAGTAT	60
		Rev	CGTCTTCTGTGTGGCTGTGATG	

### Confocal Microscopy for Transferrin Uptake

Splenocytes were treated with I) MDV sup (10%) II) Vaccine sup (10%) III) CEF mock IV) 265L sup (10%) for 24h and briefly cultured with media containing transferrin from human serum, Alexa Fluor™ 647 conjugate (Life Technologies, Warrington, UK) for 20 min before fixing and staining nuclei with 4’,6’-diamidino-2-phenylindole (DAPI). Slides were imaged on Leica SP2 laser-scanning confocal microscope and the results were analysed using ImageJ software.

### Statistical Analysis

Data are represented as mean ± SEM. The transferrin data are analysed using ANOVA Kruskal-Wallis test with *post hoc* Dunn’s multiple comparison analysis. The mean rank of mock sample was 71.95 and alpha 0.05. The proliferation data were analysed using ordinary one-way ANOVA with *post hoc* Dunnett’s multiple comparison test with single pooled variance. GraphPad prism software (San Diego, CA) was used for performing statistical analysis. Results were considered statistically significant at a P value of <0.05 (*).

## Results

### MDV-Induced COX-2/PGE2 Pathway Impairs T Cell Proliferation *In Vivo*

**Figure 1:**
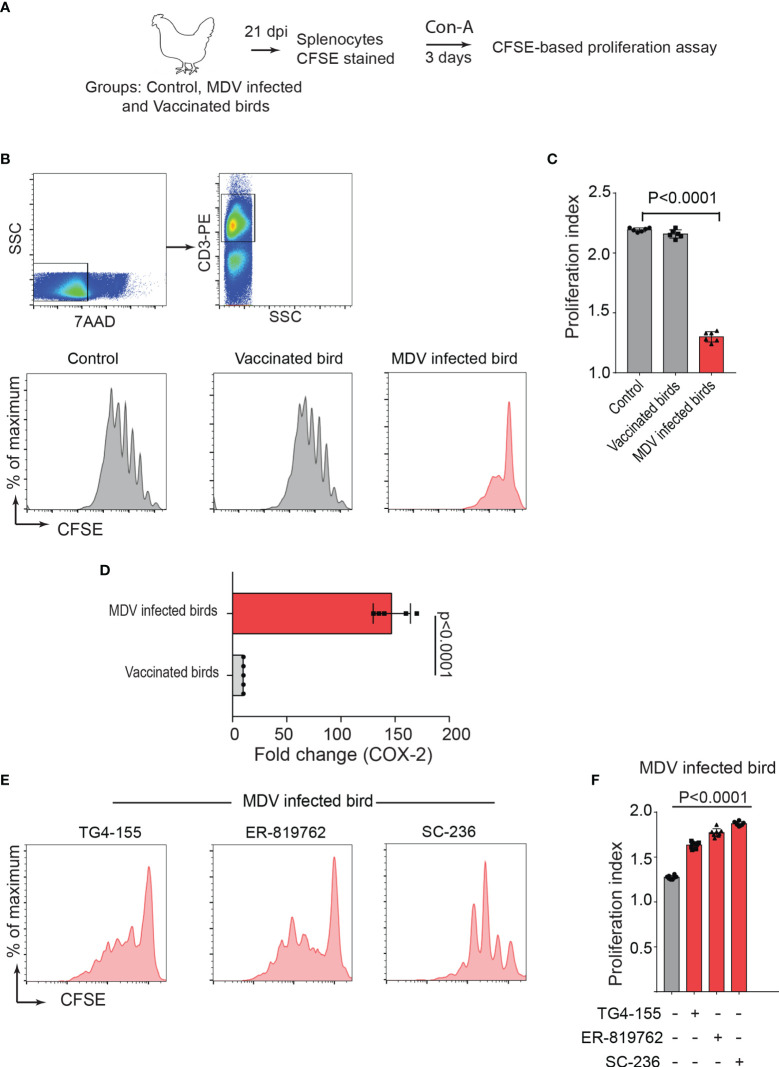
MDV infection upregulates COX-2 expression and impairs T cell proliferation. **(A)** Graphical presentation for experimental setup for *ex vivo* proliferation assay: Splenocytes, harvested at 21 dpi from control, MDV infected (RB1B, 1,000 pfu/dose), and vaccinated birds (Rispens-CVI988, 1,000 pfu/dose), were stained with CFSE and stimulated with Con-A (5 µg/ml) for 3 days. **(B)** Representative dot plot and histograms showing the gating strategy used in acquisition and analysis of CFSE based proliferation data. The proliferation of 7AAD^-^ (live cells) CD3^+^ T cells was analysed by flow cytometry. **(C)** Proliferation index showing proliferation in CD3+ T cells from control, MDV infected and vaccinated birds. **(D)** qRT-PCR data showing fold change in mRNA level for *COX-2* gene in MDV infected and vaccinated birds which was calculated over the control birds. **(E)** Representative histograms and corresponding **(F)** proliferation index showing proliferation in CD3+ T cells from MDV infected birds stimulated with Con-A in presence of the chemical inhibitors: TG4-155 (4 μM), ER-819762 (8 μM) and SC-236 (5 μg/mL). Grey bars: No significant difference between mock and treatment and red bars: significant difference or restoration to non-significant level between mock and treatment group. Dot plot and histograms are representative of proliferation data from six individual infected birds. Each dot in proliferation index represents the average of three biological replicates from six individual chickens. Error bar represents the mean ± standard deviation. Statistical significance was estimated as *p* value calculated by ANOVA test.

One-day old Line P2a chickens (MHC B^19^ haplotype) were administered with non-infected chicken embryo fibroblast cells (CEF) (control group), CEFs infected with virulent strain RB1B (MDV infected group) or CEFs infected with vaccine strain Rispens-CVI988 (vaccinated group). Since MDV-induced Treg cell expansion had been previously reported only at day 21 post infection (22), we initially examined the role of MDV-induced PGE2 on impairment of T cell proliferation at 21 days post infection (dpi). T cell proliferation in response to Concanavalin-A (ConA) stimulation, was analysed *in vitro* using CFSE-based proliferation assay (**Figure 1A**). The results demonstrated a significant reduction in CD3+ T cell proliferation in the MDV-infected chickens, while no significant difference was observed between the vaccinated and control groups (**Figures 1B, C**). These results indicated that infection of chickens with the virulent strain of MDV impaired CD3+ T cell proliferation. Our recent report had demonstrated that MDV infection of CEF activates the COX-2/PGE2 pathway *in vitro* (15), however, it was unclear whether the vaccine strain of MDV can activate this pathway. To examine whether virulent and vaccine strains MDV can activate the COX-2/PGE2 pathway *in vivo*, the expression levels of COX-2 gene were analysed in splenocytes of chickens within the different experimental groups using RT-PCR. The results demonstrate that the inoculation of virulent MDV, but not vaccine strain of MDV, upregulates COX-2 gene expression in the splenocytes by over 150-fold (**Figure 1D**). To determine the role of COX-2/PGE2 pathway in the MDV-induced T cell dysfunction, the splenocytes were treated with chemical inhibitors of the COX-2/PGE2 pathway, TG4-155 (EP2 blocker), ER-819762 (EP4 blocker) and sc-236 (COX-2 inhibitor), and T cell proliferation was analysed using CFSE-based proliferation assays. All the COX-2/PGE2 inhibitors restored CD3+ T cell proliferation in MDV-infected chickens (**Figures 1E, F**), while these inhibitors did not alter proliferation rate in the control groups, indicating that MDV-induced COX-2/PGE2 pathway suppresses T cell proliferation in an EP2 and EP4 dependent manner.

### MDV Downregulates IL-2 Expression and Transferrin Uptake in a PGE2 Dependent Manner

The main identified mechanisms involved in inhibition of human T cell proliferation by PGE2 are downregulation of interleukin (IL)-2, IL-2 receptors, and transferrin uptake (23, 30). To determine the mechanisms involved in the MDV-induced impairment of T cell proliferation, the effects of PGE2 produced by MDV-infected chicken cells on (a) chicken IL-2 gene expression using RT-PCR, (b) the expression of alpha chain of the high-affinity IL-2 receptor (CD25) expression using flow cytometry and (c) transferrin uptake using confocal microscopy were analysed.

**Figure 2:**
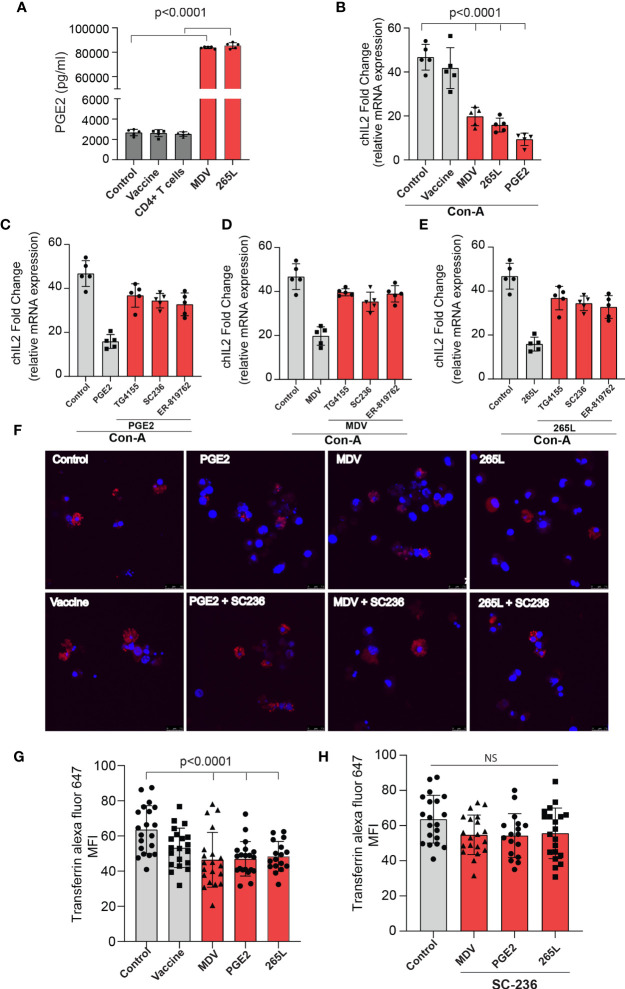
MDV infected CEFs and 265L tumour cells produce high PGE2 level resulting in downregulation of chIL2 and transferrin uptake in CD3+ T cells. Culture supernatants were harvested from control CEF (non-infected),CEF infected with virulent MDV (MDV supernatant) or vaccine strain of MDV (vaccine supernatant) at 72 hrs post-infection. Culture supernatant harvested from CD4+ T cells of naïve splenocytes and MDV-transformed CD4+ T cells (265L supernatant). **(A)** The levels of PGE2 were determined using an ELISA assay. **(B)** chIL2 expression level was determined in Con-A stimulated splenocytes treated with PGE2 (10 µg/ml), or the supernatants as described above using qRT-PCR, and fold change are shown. Inhibition of chIL-2 expression by **(C)** PGE2, **(D)** MDV supernatant, **(E)** 265L supernatant was rescued in the presence of the inhibitors of the COX-2/PGE2 pathway; TG4-155 (4 μM), ER-819762 (8 μM) and SC-236 (5 μg/mL). **(F)** Representative confocal images showing uptake of transferrin (red color) by CD3+ T cells in the Con-A stimulated splenocytes treated with control supernatant, PGE2, MDV supernatant, vaccine supernatant or 265L supernatant using confocal microscopy. **(G)** Graph shows mean fluorescent intensity (MFI) of transferrin uptake using confocal microscopy. **(H)** Bar graph shows the effects of a COX-2 inhibitor (SC-236) on transferrin uptake in CD3+ T cells treated with PGE2, MDV supernatant or 265L supernatant using confocal microscopy. Graphs are representative of three independent experiments, each performed with three biological replicates. Grey bars are used in the experimental groups in which no significant difference are found, while red bars are used to represent significant differences. Each dot in ELISA represents the average of three biological replicates from six individual culture groups. Error bar represents the mean ± standard deviation. Statistical significance was estimated as *p* value calculated by ANOVA test. NS, not significant.

In brief, cell culture supernatants isolated from mock (control), virulent MDV (MDV supernatant.; RB1B strain of MDV; 100pfu) and vaccine strain (Vaccine supernatant; Rispens-CVI988 strain of MDV; 100 pfu) infected CEF were collected at 72 hpi and the levels of PGE2 were analysed using a competitive ELISA assay for PGE2 (**Figure 2A**). In addition, PGE2 levels in cell culture supernatant from non-stimulated purified CD4+ T cells, 265L cells (265L supernatant), an MDV-transformed CD4+ T cell line, were also determined. The results demonstrated that the MDV supernatant, but not the vaccine supernatant, had high levels of PGE2 (~8 ng/ml) (**Figure 2A**), indicating that there is an association between pathogenicity and release of PGE2 by the infected cells. To attribute impairment of T cell proliferation to the MDV-induced PGE2, chicken splenocytes were treated with either recombinant PGE2 or the cell culture supernatants and subsequently the cells were stimulated with Con-A. Upregulation of chIL-2 expression levels were determined in the Con-A stimulated cells and fold change was calculated based on IL-2 levels in unstimulated cells using RT-PCR. The results demonstrate that splenocytes treated with PGE2 or the soluble factor(s) released by the MDV-infected cells, but not the cells infected with the vaccine strain, downregulates chIL2 expression levels compared to splenocytes treated with the cell culture supernatant from the control groups (**Figure 2B**). The inhibitory effects of recombinant PGE2 (**Figure 2C**), MDV supernatant (**Figure 2D**) or 265L supernatant (**Figure 2E**) on chIL2 expression were rescued by treatment of splenocytes with chemical inhibitors of EP2 (TG4-155), EP4 (ER-819762) or COX-2 (SC-236). The chemical inhibitors did not alter IL-2 expression in control groups.

**Figure 3:**
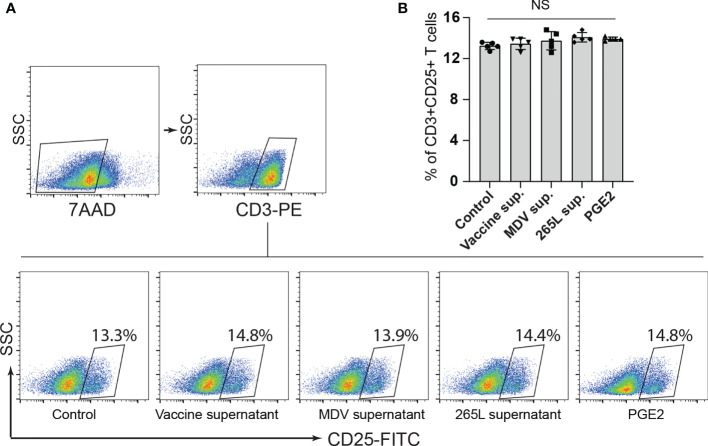
Expression of CD25^+^ on CD3^+^ T cells remained unaffected by PGE2 treatment. CD25 surface expression on CD3+ T cells were quantified in splenocytes stimulated with Con-A (5 µg/ml) in presence of cell culture media containing either recombinant PGE2 (5 µg/ml), the control supernatants, MDV supernatant, vaccine supernatant or the 265L supernatant. **(A)** Dot plot **(B)** bar graph showing percentage of CD3^+^CD25^+^ T cells. Graph is representative of three independent experiments, each performed with three biological replicates per treatment. Each dot in ELISA represents the average of three biological replicates from six individual culture groups. Grey bars represent the experimental groups with no significant difference. Error bar represents the mean ± standard deviation. Statistical significance was estimated as *p* value calculated by ANOVA test. NS, not significant.

Next, we assessed if the transferrin uptake by the PGE2-treated splenocytes is affected. Our data demonstrated a significant reduction in transferrin uptake in the cells treated with recombinant PGE2 or the soluble factors released by MDV-infected cells. In contrast, splenocytes treated with the vaccine supernatant did not inhibit transferrin uptake (**Figures 2F, G**). The uptake of transferrin in splenocytes was rescued and restored by treatment of splenocytes with chemical inhibitor of COX-2 (SC-236) (**Figure 2H**). The chemical inhibitor did not alter transferring uptake in the control group. Altogether, the results demonstrated that MDV downregulated IL-2 expression and inhibited transferrin uptake in a PGE2 dependent manner. In contrast, there was no effect on percentages (**Figures 3A, B**) of CD3+ T cells expressing CD25 molecules.

### MDV Impairs T Cell Proliferation in a COX-2/PGE2 Dependent Mechanism *In Vitro*

**Figure 4:**
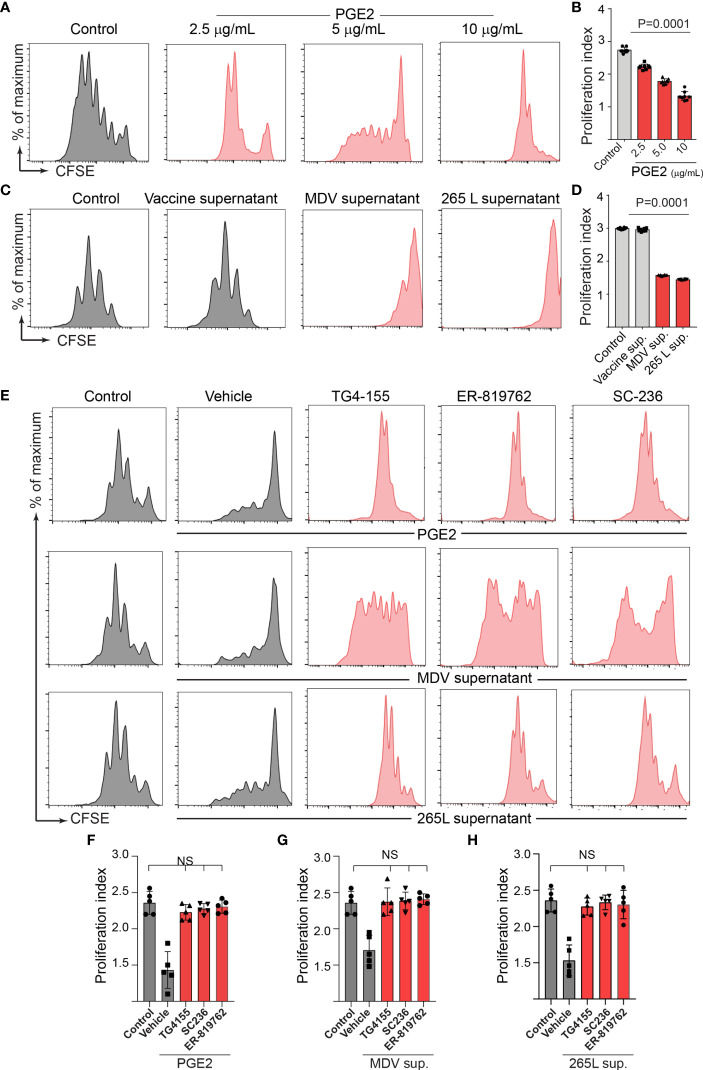
MDV infection drives PGE2/COX-2 dependent downregulation of T cell proliferation *in vitro*. Splenocytes were harvested from naïve birds and stained with CFSE and then they were stimulated with Con-A (5 µg/ml) in presence of PGE2, control supernatant, MDV supernatant, Vaccine supernatant, or 265L supernatant with or without the chemical the inhibitors of the PGE2-COX-2 pathway. After 3 days of culture, T cell proliferation was analysed by flow cytometry. **(A)** Representative histograms and corresponding **(B)** proliferation index showing dose-dependent effect of recombinant PGE2 (2.5, 5 and 10 µg/ml) on *in vitro* proliferation of CD3+ T cells. **(C)** Representative histograms and corresponding **(D)** proliferation index showing effect of control supernatant, MDV supernatant, Vaccine supernatant, or 256L supernatant on *in vitro* proliferation of CD3+ T cells. **(E, G)** Representative histograms and corresponding **(F, H)** proliferation index showing inhibitory effect of the chemical inhibitor: TG4-155 (4 μM), ER-819762 (8 μM) and SC-236 (5 μg/mL). Dot plot and histograms are representative of proliferation data from six individual birds. Grey bars are used in the experimental groups in which no significant difference are found, while red bars are used to represent significant differences. Each dot in proliferation index represents the average of three biological replicates from six individual chickens. Error bar represents the mean ± standard deviation. Statistical significance was estimated as *p* value calculated by ANOVA test. NS, not significant.

Series of *in vitro* experiments were performed to determine the role of MDV-induced PGE2 in the impairment of T cell proliferation. Splenocytes from non-infected naïve Rhode Island Red (RIR) chickens were treated with different concentrations of recombinant PGE2, the MDV supernatant, the vaccine supernatant or the 265L supernatant in presence or absence of the chemical inhibitors of EP2 (TG4-155), EP4 (ER-819762) or COX-2 (SC-236). T cell proliferation was determined using a CFSE-based proliferation assay in response to Con-A stimulation. Recombinant PGE2 (2.5, 5 and 10 ug/ml) suppressed CD3+ T cell proliferation in a dose-dependent manner (**Figures 4A, B**). Similarly, the MDV supernatant or the 265L supernatant significantly reduced CD3+ T cell proliferation. In contrast, treatment of splenocytes with the vaccine supernatant did not reduce their ability to proliferate *in vitro* (**Figures 4C, D**). Beyond that, the results showed that the chemical inhibitors of the COX-2/PGE2 pathway; TG4-155 (EP2 blocker), ER-819762 (EP4 blocker) and SC-236 (COX-2 inhibitor) inhibited impairment of T cell proliferation induced by the recombinant PGE2 (**Figures 4E, F**), the soluble factors released from MDV-infected cells (**Figures 4E, G**) and soluble factors released from MDV-transformed T cell lines (265L sup.) (**Figures 4E, H**). The chemical inhibitors did not alter T cell proliferation in the control groups. Taken together, the results demonstrated that PGE2 released by the MDV-infected cells impaired T cell proliferation in a COX-2/PGE2 dependent manner *in vitro*.

### The MDV-Induced COX-2/PGE2 Pathway Impaired T Cell Proliferation by Directly Affecting CD3+ T Cells

**Figure 5:**
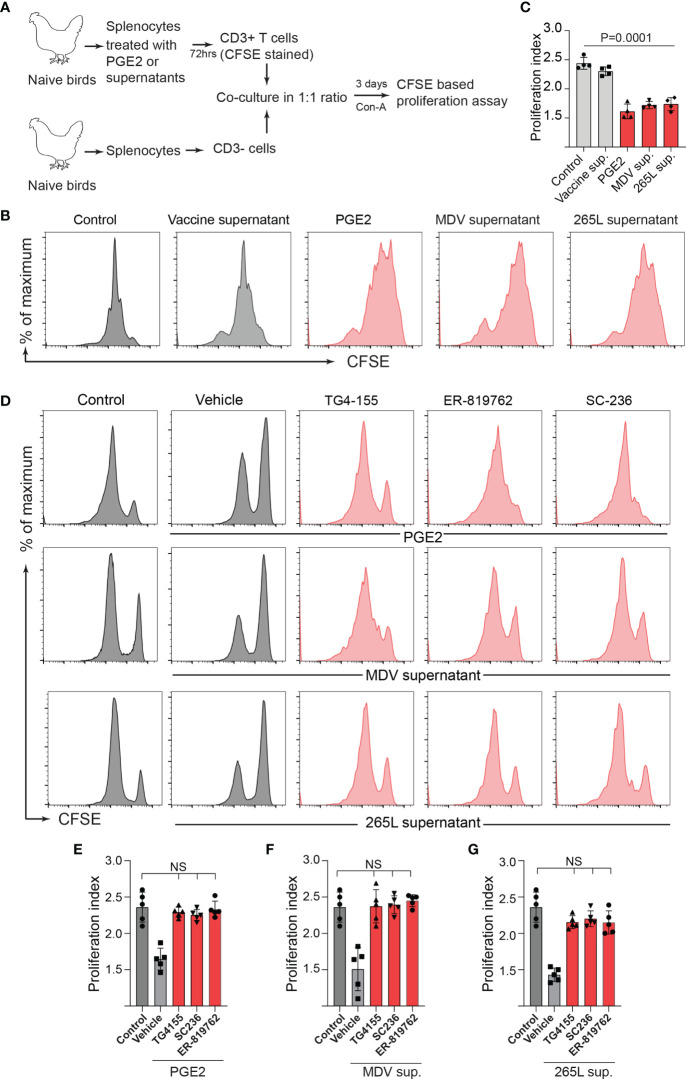
Treatment of splenocytes with PGE2/MDV sup/265L sup induced PGE2-COX-2 pathway drives dysfunction of CD3^+^ T cells. **(A)** Splenocytes were cultured for 3 days in media containing recombinant PGE2 (5 µg/ml), control supernatant, MDV supernatant, vaccine supernatant or 265L supernatant. The isolated CD3^+^ T cells were CFSE stained and co-cultured with freshly isolated CD3 negative cells. The co-culture was stimulated with Con-A (5 µg/ml) in presence or absence of the chemical inhibitors of the PGE2-COX-2 pathway. After 3 days of co-culture, T cell proliferation was analysed by flow cytometry. **(B)** Representative histograms and corresponding **(C)** proliferation index. **(D)** Representative histograms and corresponding **(E–G)** proliferation index showing *in vitro* proliferation in CD3^+^ T cells from splenocytes cultured with PGE2, MDV Supernatant and 265L supernatant in presence of the chemical inhibitors: TG4-155 (4 μM), ER-819762 (8 μM) and SC-236 (5 μg/mL), respectively. Data is representation of three independent experiments, each performed with three biological replicates per treatment. Grey bars are used in the experimental groups in which no significant difference are found, while red bars are used to represent significant differences. Each dot represents the data from individual chicken and the graph represents the mean ± standard deviation of five independent replicates from individual chickens. Statistical significance was estimated by *p* value calculated by ANOVA test. NS, not significant.

To examine whether the MDV-induced COX-2/PGE2 pathway impaired T cell proliferation by directly affecting CD3+ T cells or indirectly *via* inhibition of other immune cells present in the splenocytes, splenocytes were treated with the recombinant PGE2 or the supernatants. After 3-day culture, CD3^+^ T cells were isolated from the treated splenocytes, CFSE-stained and then cocultured with the purified fresh untreated CD3^neg^ cells isolated from naïve chickens in presence or absence of the chemical inhibitors of the COX-2/PGE2 pathway. T cell proliferation was analysed using CFSE-based proliferation assay upon 72 hrs CoA stimulation (**Figure 5A**). The results demonstrated that the isolated CD3^+^ T cells which were treated with the recombinant PGE2, the MDV supernatant or the 265L supernatant, but not the vaccine supernatant, have lower proliferation index compared to the control T cells (**Figures 5B, C**). The inhibitory effects of the recombinant PGE2 (**Figures 5D, E**), the MDV supernatant (**Figures 5D, F**) or the 265L supernatant (**Figures 5D, G**) were rescued by the chemical inhibitors of the COX-2/PGE2 pathway. Taken together, our *in vitro* experiments indicated that the MDV-induced COX-2/PGE2 pathway impaired T cell proliferation by directly affecting CD3^+^ T cells.

**Figure 6:**
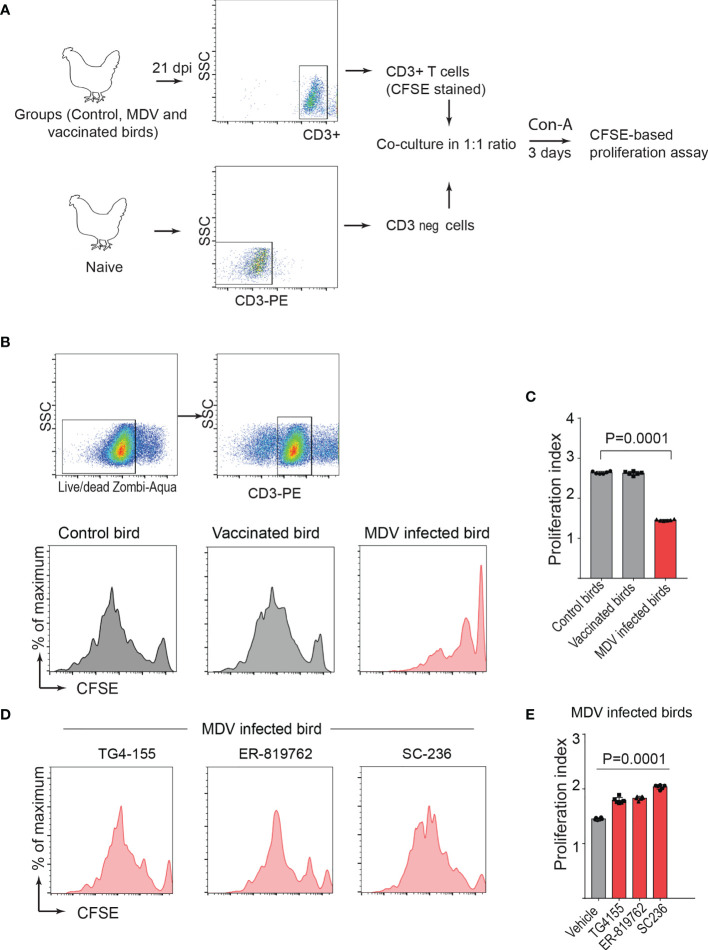
PGE2-COX-2 drives dysfunction of CD3^+^ T cells in MDV infected chickens. **(A)** CD3^+^ T cells were isolated from splenocytes harvested at 21 dpi from control (n=5), MDV infected (n=5) and vaccinated birds (n=5) and stained with CFSE before co-culturing with CD3 negative T cells isolated from splenocytes of naïve birds. The co-culture was stimulated with Con-A (5 µg/ml) in presence or absence of the chemical inhibitors of PGE2-COX-2 pathway. After 3 days of co-culture, T cell proliferation was analysed by flow cytometry. **(B, D)** Representative histograms showing proliferation in CD3+ T cells from the control, vaccinated birds, and MDV infected birds with or without the chemical inhibitors: TG4-155 (4 μM), ER-819762 (8 μM) and SC-236 (5 μg/mL) and corresponding **(C)** proliferation index from the experimental groups of birds. **(E)** Proliferation index for CD3^+^ T cells from the MDV infected birds in presence of the chemical inhibitors of the PGE2-COX-2 pathway. Each dot represents the data from individual chicken and the graph represents the mean ± standard deviation of five independent replicates from individual chickens. Grey bars are used in the experimental groups in which no significant difference are found, while red bars are used to represent significant differences. Statistical significance was estimated by *p* value calculated by ANOVA test.

To confirm this in the MDV-infected chickens, one-day-old chickens were inoculated with the virulent MDV (RB1B: 1,000 pfu/dose; n=5), vaccine strain of MDV (Rispens-CVI988; 1,000 pfu/dose; n=5) or mock-infected (non-infected CEF; n=5) *via* intra-abdominal route. Splenocytes were isolated from the experimental groups at 21 dpi, and CD3^+^ T cells were purified from the isolated splenocytes. In parallel, CD3^neg^ cells, from non-infected naïve chickens, were co-cultured with the isolated CD3^+^ T cells from the MDV-infected groups (**Figure 6A**) in presence or absence of the chemical inhibitors of the COX-2/PGE2 pathway. CD3^+^ T cell proliferation was analysed, 3 days after Con-A stimulation, using CFSE-based proliferation assay (**Figure 6B**). The results demonstrated that CD3^+^ T cells isolated from the MDV infected chickens, but not the vaccinated chickens, were impaired and the coculture with naive CD3^neg^ cells did not rescue CD3^+^ T cell proliferation (**Figures 6B, C**). Furthermore, the results showed that the chemical inhibitors of the COX-2/PGE2 pathway rescued T cell proliferation in this system (**Figures 6D, E**).

**Figure 7:**
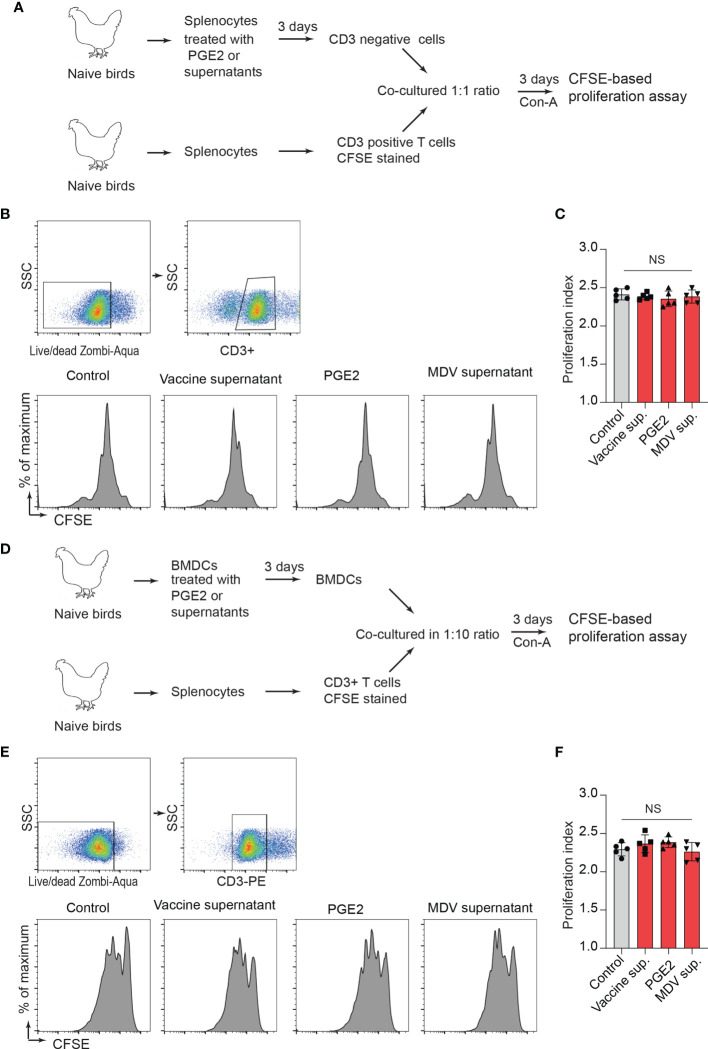
PGE2/MDV supernatant directly affected CD3+ T cells and impaired CD3^+^ T cells is independent of CD3 negative T cells and BMDCs. **(A)** CD3 negative cells were isolated from splenocytes cultured for 3 days in media containing recombinant PGE2 (5 µg/ml), the control supernatant, MDV supernatant, vaccine supernatant or 265L supernatant. The isolated CD3 negative cells were co-cultured with CFSE stained CD3^+^ T cells isolated from naive birds in presence of Con-A (5 µg/ml). After 3 days of co-culture, T cell proliferation was analysed by flow cytometry. **(B, C)** Representative histograms and corresponding **(C)** proliferation index showing *in vitro* proliferation of CD3+ T cells from the experimental groups. **(D)** BMDCs were cultured for 3 days with media containing recombinant PGE2 (5 µg/ml), the control supernatant, MDV supernatant, vaccine supernatant or 265L supernatant. The treated BMDCs were harvested, washed, and cultured with CFSE stained CD3^+^ T cells isolated from naïve birds in fresh medium containing Con-A (5 µg/ml) for 3 days. **(E)** Representative histograms and corresponding **(F)** proliferation index for CD3^+^ T cells from the different experimental groups. Data is representation of three independent experiments, each performed with three biological replicates per treatment. Each dot represents the data from individual chicken and the graph represents the mean ± standard deviation of five independent replicates from individual chickens. Grey bars are used in the experimental groups in which no significant difference are found, while red bars are used to represent significant differences. Statistical significance was estimated by *p* value calculated by ANOVA test. NS, not significant.

Next, a series of experiments were performed to determine whether treatment of CD3^neg^ cells with the recombinant PGE2 or the supernatants prior to co-culture with CD3^+^ T cell can inhibit CD3^+^ T cell proliferation. *In vitro*, splenocytes were treated with the recombinant PGE2, the MDV supernatant, the vaccine supernatant or the 265L supernatant for 72 hrs. After 3-day culture, CD3^neg^ cells were purified and co-cultured with CFSE-stained fresh CD3^+^ T cells (from naïve birds) in presence of Con-A for 3 days, and T cell proliferation was analysed using flow cytometry (**Figure 7A**). The results demonstrated that CD3^neg^ cells treated with PGE2, or the soluble factors released from the MDV-infected cells were unable to suppress CD3^+^ T cell proliferation *in vitro* (**Figures 7B, C**). To confirm these data using professional APCs, bone marrow derived dendritic cells (BMDCs) were generated *in vitro* and were treated with PGE2, or the soluble factors released by the MDV-infected cells for 3 days. Then, the treated BMDCs were co-cultured with freshly isolated CFSE-labelled naïve CD3^+^ T cells and Con-A-induced T cell proliferation was examined using flow cytometry (**Figure 7D**). The results demonstrated that BMDCs treated with PGE2 or the soluble factors from the MDV-infected cells failed to suppress T cell proliferation (**Figures 7E, F**). To confirm these findings in the MDV infected chickens, CD3^neg^ cells were isolated from splenocytes of chickens infected with the virulent strain of MDV or mock control group at 21 dpi and were co-cultured with CFSE-stained CD3^+^ T cells isolated from non-infected naïve chickens in the presence of Con-A (**Figure 7A**). The results demonstrated that CD3^neg^ cells isolated from the MDV-infected chickens were unable to suppress T cell proliferation (**Figures 7B, C**). Altogether, these results indicated that the MDV-induced COX-2/PGE2 pathway directly affect CD3+ T cells and suppress MDV-induced impairment of T cell proliferation.

### Administration of COX-2 Inhibitor Rescued T Cell Proliferation in the MDV Infected Chickens

**Figure 8:**
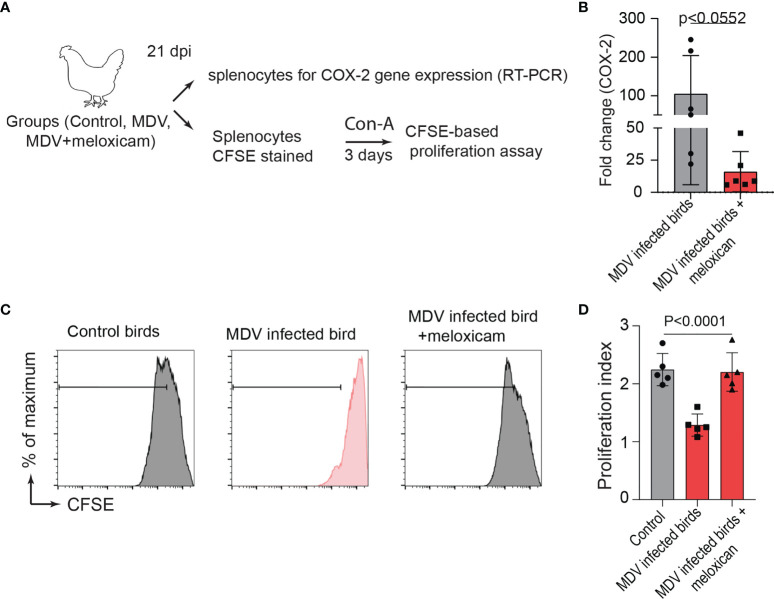
Administration of meloxicam rescues T cell proliferation in MDV infected chickens. **(A)** Schematic diagram showing the experimental groups; non-infected mock controls, MDV infected birds (RB1B, 1,000 pfu/dose), and MDV infected birds that received daily oral administration of meloxicam. **(B)** COX-2 expression levels in splenocytes of the experimental groups were analysed using RT-PCR assay. CFSE-labelled splenocytes were stimulated with Con-A and cultured for 3 days *in vitro* to assess T cell proliferation using flow cytometry. **(C)** Representative histograms and corresponding **(D)** proliferation index showing *ex vivo* proliferation of CD3+ T cells in mock control and MDV infected with and without meloxicam treated birds. Grey bars are used in the experimental groups in which no significant difference are found, while red bars are used to represent significant differences. Each dot represents the data from individual chicken and the graph represents the mean ± standard deviation of five independent replicates from individual chickens. Statistical significance was estimated by *p* value calculated by ANOVA test.

To examine the role of the MDV-induced COX-2/PGE2 pathway in the dysfunction of T cell proliferation, MDV-infected chickens were orally treated (daily administration until 21 dpi) with meloxicam (a nontoxic COX-2 inhibitor which is used in clinical cases in birds). At day 21 post infection, splenocytes were isolated and expression of COX-2 gene and CD3+ T cell proliferation were analysed in the experimental groups using RT-PCR and a CFSE-based proliferation assays, respectively (**Figure 8A**). Interestingly, the oral administration of meloxicam in the MDV-infected chickens downregulated COX-2 expression levels (**Figure 8B**) and rescued CD3^+^ T cell proliferation (**Figures 8C, D**).

## Discussion

Foxp3-like genes has not been identified in the chicken genome (31) and thus CD25 and membrane bound TGF-beta are used to identify chicken Treg cells (22, 32). In MDV infection, there is a shift towards TGF-beta+ CD4+ T cells while the frequencies of CD4+CD25+ T cells are unchanged (22). MDV infection is characterized by the induction of immunosuppression (33, 34), and a reduction in proliferation of non-transformed T cells. This impairment has been attributed to activation of immunomodulatory molecules by tumour associated macrophages (35), increased production of arginase by chicken macrophages from MDV infected chickens (36) and expansion of TGF-beta+ Treg cells expressing inhibitory molecules such as CTLA4 and PD1 (22). Here, the role of this COX-2/PGE2 pathway activation in MDV-induced immunosuppression and specifically the impairment of T cell proliferation during virus infection is examined at day 21 post infection. *In vitro* infection of chicken embryonated fibroblasts with virulent strain of MDV activates COX-2/PGE2 pathway which is involved in efficient replication of MDV *via* Ep2 and EP4 receptors which are upregulated in MDV infected cells (15). It is speculated that PGE2 may contribute to MDV replication by inhibiting other pathway(s) (*e.g.* NO production, type I IFN production etc.) which potentially can restrict MDV replication. It seems that dynamic of Rispen-CVI988 replication is different than the virulent MDV (37), as the vaccine strain does not activate COX-2/PGE2 pathway and thus does not require PGE2 for its replication. It is possible, but not proven, that infection of cells with Rispens-CVI988 does not activate these inhibitory pathways. COX-2 is considered as an oncogene in mammalian models, and it is possible that COX-2 is activated by MDV genes which have different sequences in MDV and Rispens-CVI998 (*e.g.* MEQ). It is noteworthy to mention that only small residues of non-structural protein 2 from highly pathogenic porcine reproductive and respiratory syndrome virus promote COX-2 expression in infected pigs leading to clinical signs of the disease (38). We are currently examining the role of MDV gene in activation of COX-2/PGE2 pathway in MDV infected cells. However, it is still unclear whether activation of the COX2/PGE2 pathway is associated with MDV pathogenesis. The results from this study indicate that there is an association between pathogenesis and activation of COX-2 which leads to impairment of T cell proliferation *in vitro* and *in vivo*.

Our result reveals that induction of the COX2/PGE2 pathway is only observed in chickens infected with virulent strain of MDV, while administration of vaccine strain of MDV (Rispens-CVI988) does not lead to this activation *in vitro* or *in vivo*. Interestingly, the results show that the COX2/PGE2 pathway activation is involved in the impairment of chicken T cell proliferation, and perhaps MDV-induced immunosuppression. Our results also demonstrate that the MDV-induced COX-2/PGE2 pathway suppresses IL 2 gene expression and transferrin uptake. Further research is required to examine whether the release of IL-2 protein is also influenced by this pathway. The effects of MDV-induced COX2/PGE2 pathway on T cell function is in accordance with results showing that PGE2 suppresses human T cell activation and proliferation *via* two distinct pathways: inhibition of transferrin receptor expression and IL 2 production (23, 39). In contrast, MDV-induced COX2/PGE2 pathway did not modulate CD25 molecule (IL-2 receptor alpha chain) which has been shown to be modulated by PGE2 on human immune cells (40, 41). Interestingly, COX2 inhibitor blocked the effects of PGE2 on T cells, this is only possible if PGE2 can activate COX-2 expression, and this can lead to further release of PGE2 (42). Many mammalian viruses activate the COX-2/PGE2 pathway and contribute to virus replication and spread (11, 43–48), however the role of virus-induced COX2/PGE2 pathway in pathogenesis and especially virus-induced immunosuppression has not been extensively studied. Many viruses may suppress T cell proliferation by inhibiting the function of antigen presenting cells (APCs). Previous results had shown that MDV infection modulates the function of APCs at day 10 post infection (35, 36). In this study, we examined T cell proliferation 21 dpi, and demonstrated that MDV-induced COX2 pathway directly affect T cell proliferation and addition of fresh APCs could not rescue T cell function. The lack of CD3+ responsiveness as a function of time (5, 10 and 21 dpi) is beyond the scope of this study. It is possible that APC populations may already have patterned the T-cells to be a sub-lineage (i.e., Treg), prior to 21 dpi. Considering that PGE2 may modulate the function of APCs (49), it was expected that impairment of T cell proliferation in MDV-infected chickens may be, at least partially, be attributed to the effects of PGE2 on APCs. Surprisingly, our results demonstrate that APCs are not involved in the MDV-induced impairment of T cell proliferation at day 21 post infection. Intriguingly, even recombinant PGE2-treated APCs failed to suppress T cell proliferation. This does not exclude the possibility that MDV-induced COX2/PGE2 activation may modulate other important functions of immune cells (*e.g.* cytokine production, phagocytosis, induction of T cell subsets, suppression of T cell cytotoxicity etc.) (49–51) which may be important in pathogenesis of viral infections. Combination therapy of COX-2 inhibitors and anti-PD1 antibodies have been used in murine models to increase the efficacy of COX-2 inhibitors in the context of T cell functions and survival during chronic viral infections in mice (17) and cattle infected with leukaemia virus (25). It has been suggested that PGE2 may upregulate PD-L1 expression, and this may suppress effector Th1 type responses (26). Antibodies against chicken PD1 and PDL1 have been generate at our institute (52), and future studies will be conducted to examine the combined effects of COX-2 inhibitors and PD1 antibodies on both viral replication, T cell function and disease progress in MDV-infected chickens.

MDV infection leads to development of CD4+ T cell lymphoma in infected chickens, thus it is possible that the MDV-induced COX2/PGE2 pathway may contribute to tumorigenesis of MDV by suppressing immune response against MDV-induced CD4+ lymphoma cells. The role of COX-2 as an oncogene and its suppression of anti-tumour immunity has been extensively studied in mammalian models, and EP4 inhibitors have been suggested in cancer therapeutic and immunotherapy approaches (53, 54). Understanding the role of MDV-induced COX2/PGE2 activation in the modulation of immune responses against viral infection and tumorigenesis may lead to the development of novel vaccination strategies. Moreover, further studies are required to examine the role COX2/PGE2 pathway in tumorigenesis of MDV and modulation of anti-tumour immunity. Therefore, it may be possible that MDV infection could be used as a model for the development of novel treatment strategies by targeting the COX2/PGE2 pathway to control viral replication, virus-induced immunosuppression, and tumorigenesis.

## Data Availability Statement

The original contributions presented in the study are included in the article/supplementary material. Further inquiries can be directed to the corresponding author.

## Ethics Statement

The animal study was reviewed and approved by Animal Welfare and Ethical Review Body at The Pirbright Institute.

## Author Contributions

Conceptualization: SB and AP. Data curation: NK, AG, and SB. Formal analysis: NK, AG, and SB. Funding acquisition: SB. Investigation: NK, AG, and SB. Methodology: NK, AG, and SB. Supervision: SB Visualization: NK and AG. Writing – original draft: NK, AG, BK, AP, and SB. Writing – review & editing: SB, BK, and AP.

## Funding

This work was supported by U.K. Research and Innovation Biotechnology and Biological Sciences Research Council Grants BBS/E/I/00001825, BBS/E/I/00007030, BBS/E/I/00007031, BB/S01506X/1, BBS/E/I/00002529, BBS/E/I/00007039, BBS/E/I/00007032, BB/N002598/1 andBB/V019031/1.

## Conflict of Interest

The authors declare that the research was conducted in the absence of any commercial or financial relationships that could be construed as a potential conflict of interest.

## Publisher’s Note

All claims expressed in this article are solely those of the authors and do not necessarily represent those of their affiliated organizations, or those of the publisher, the editors and the reviewers. Any product that may be evaluated in this article, or claim that may be made by its manufacturer, is not guaranteed or endorsed by the publisher.
